# Health monitoring of finishing pigs by secondary data use – a longitudinal analysis

**DOI:** 10.1186/s40813-021-00197-z

**Published:** 2021-02-24

**Authors:** Julia Grosse-Kleimann, Heiko Plate, Henning Meyer, Hubert Gerhardy, Corinna Elisabeth Heucke, Lothar Kreienbrock

**Affiliations:** 1 Department of Biometry, Epidemiology and Information Processing, WHO Collaborating Centre for Research and Training for Health in the Human-Animal-Environment Interface, University of Veterinary Medicine Hannover, Foundation, Buenteweg 2, 30559 Hanover, Germany; 2 VzF e.V., Association for Promoting Farming Economics, Uelzen, Germany; 3 MSG, Marketing Service Gerhardy, Garbsen, Germany

**Keywords:** Animal welfare, Antimicrobial treatment, Meat inspection, Performance data

## Abstract

**Background:**

In Germany, animal welfare has become an increasingly important issue. Since 2006, German legislation demands self-monitoring of animal welfare by farmers, but there is a lack of prescribed indicators for governmental monitoring. Since recording of the health status through examinations on individual farms requires many resources, secondary data use is obvious. Therefore, this study deals with the overall evaluation and utilization of existing production data from the German pork production. Performance data and information on antibiotic usage and meat inspection were used for a benchmarking system of animal health in finishing pigs.

**Results:**

Seven health scores and one total score were evaluated for 184 finishing pig herds on semi-annual basis between July 2017 and June 2019, based on the health indicators mortality, average daily gain, feed conversion ratio, treatment frequency, respiratory lesions, exterior lesions and animal management. In preparation, the selected health indicators were brought to the same scale and skewed data were transformed to build scores (MOR, ADG, FCR, TF, RESP, EXT and MANG). A differentiated analysis was carried out for three classes of initial body weight regarding to farmers’ fattening management strategies.

**Conclusions:**

The present study shows that existing production data of German finishing pigs are usable for welfare monitoring. However, preparatory editing steps are crucial. The total score can only be an estimate of health status because partly bad or good performance could be disguised. It has also been demonstrated, that relative benchmarking is suitable for depicting temporary fluctuations in the investigated collective.

**Supplementary Information:**

The online version contains supplementary material available at 10.1186/s40813-021-00197-z.

## Background

Animal welfare is an issue that goes along with livestock farming in Germany for decades. Nevertheless, in the last couple of years, farm animal health and welfare have substantially attracted political and social attention as consumers demand transparency on conditions under which food-producing animals are kept [1, 2]. Since 2006, the eleventh paragraph of the German Animal Protection Law prescribes the gathering and assessment of suitable animal-related items by all livestock keepers in a programme of self-monitoring [3]. Different stakeholders specified several indicators [4–7], but a mandatory set of suitable items as well as a general scientific evaluation are still missing. Furthermore, the term “animal welfare” was not clearly defined which leads to a lack of harmonisation and validation of data capturing because interpretation and implementation differ. The EFSA approach to define welfare describes two groups of indicators [8]: those predicting animal welfare directly or indirectly (animal-based measures), and those having an influence on animal welfare (non-animal-based measures or resource- and management-based measures). Another definition from the Brambell Report in 1965 [9] describes welfare with the help of five freedoms: freedom from hunger and thirst (I), discomfort (II), pain, injury and disease (III), fear and distress (IV) and the possibility to express normal behaviour (V). Following this approach, physical health is a major part of welfare (freedoms I and III), in addition to behavioural and mental aspects (freedoms II, IV and V). As there are only few methods to measure latter ones, whereas health measures are well established, this paper focusses on physical and biological animal-based measures as health indicators.

However, establishing the health status for each individual farm is very costly and time consuming. Therefore, this study deals with the use of already existing data from the German pork production chain for health monitoring and a suitable benchmarking system. Herd-specific data of 205 finishing pig farms for a time-span of 2 years from public or private databases were merged and analysed. We had both production data, levied within the frame of quality assurance and consulting, and mandatory data concerning antibiotic usage and meat inspection. For Germany, this is a unique approach because there is no official or governmental monitoring programme dealing with a multiple set of indicators [1].

## Material and methods

### Study design

The study was carried out in the frame of the project “Multivariate Assessment of Animal Welfare through Integrative Data Collection and Validation of Welfare Indicators in Finishing Pigs” (MulTiViS), which was supported by the German Federal Ministry of Food and Agriculture. The project was launched in April 2017 by a consortium of the University of Veterinary Medicine Hannover (“Tierärztliche Hochschule Hannover”, TiHo), the Swine Health Service of the Chamber of Agriculture in Lower Saxony (“Schweinegesundheitsdienst”, SGD), the swine service provider VzF (registered association) (VzF) and Marketing Service Gerhardy (MSG). All pig herds under study were advised by VzF and were participants of the “QS Quality Scheme for Food” (QS). This scheme covers approximately 95% of the German pig husbandry [10] and includes, among others, a monitoring programme for antibiotic usage and a data collection of results of official meat inspection at slaughter.

Out of different production directions, we decided to put the focus of this study on the finishing phase, as there is most reliable data available. In addition to this, the aim of the project was to create a benchmarking system which is adapted to fluctuation in time. For this reason, the given data was acquired and analysed semi-annual to consider temporal changes in farm management.

### Study collective

Initially, a total of 205 commercial finishing pig herds was included in the study, after the farmers were asked for their written declaration of consent to participate voluntarily. As some farmers have withdrawn their consent while project duration and some data were supplied incompletely, ultimately, 184 pig units remained for further analyses. The farms of the study collective were located in the northeast of Lower Saxony, Germany, and had a mean size of 1132 (± 555) finishing pig places (FP). Most of the farmers constantly purchased pigs that were bred in Germany (78.8%), whereas the others were steadily of Danish (4.9%) or Dutch (3.3%) origin or varied over the time (13.0%). The initial body weight (IBW) was between 6.5 kg and 47.6 kg (29 ± 5.4 kg) and live weight at slaughtering was on an average 122.6 (± 2.8) kg. As IBW is based on farmers’ management decisions, some performance criteria were impacted by the IBW, e.g. mortality, average daily gain, feed conversion ratio or antibiotic usage (Fig. 2). Hence, we decided to stratify further analyses by three different classes of piglet IBW:

1. light: < 24 kg
2. medium: 24–33,5 kg
3. heavy: > 33,5 kg

A t-test was performed to check statistical significance of the difference between the three classes in important health indicators. Previously, F-test for homogeneity of variances determined whether normal t-test or Satterthwaite-t-test should be used. A *p*-value below 0.05 in F-test indicates heterogeneous variances and leads to Satterthwaite-t-test.

### Data sources

For health monitoring of finishing pigs, three different data sources from several stages of the supply chain were selected:

1. **Farm specific production data (PD)**, including variables of biological and economic performance, i.e., mortality, average daily gain or feed conversion ratio. This information came from routine farm visits that were conducted semi-annually by VzF in the context of advising service. The documentation of the number of farm animals that died or were culled is also regulated by law in Germany [11].
2. **Application and delivery forms of antibiotic usage (AB)**, including information about the number of treated animals, duration of treatment and number of active substances. These data were also acquired via QS within the scope of the antibiotics monitoring programme. In Germany, the documentation of antimicrobial therapy in farmed animals is laid down by legislation in the 16th amendment of the German Medicinal Products Act [12].
3. **Diagnostic data from slaughter (SL)**, containing results of official meat inspection at the abattoirs. These data were provided by QS and came from a specific scheme that looks for 13 carcass and organ lesions of pigs at slaughter [13]. The classic findings (pneumonia, pleurisy, pericarditis and liver milk spots) were supplemented by new ones, initially on a voluntary basis, in 2016 and made mandatory from 2018 [14]. Routine post-mortem meat inspection in Germany is carried out according to EU regulation [15].

**Fig. 1:**
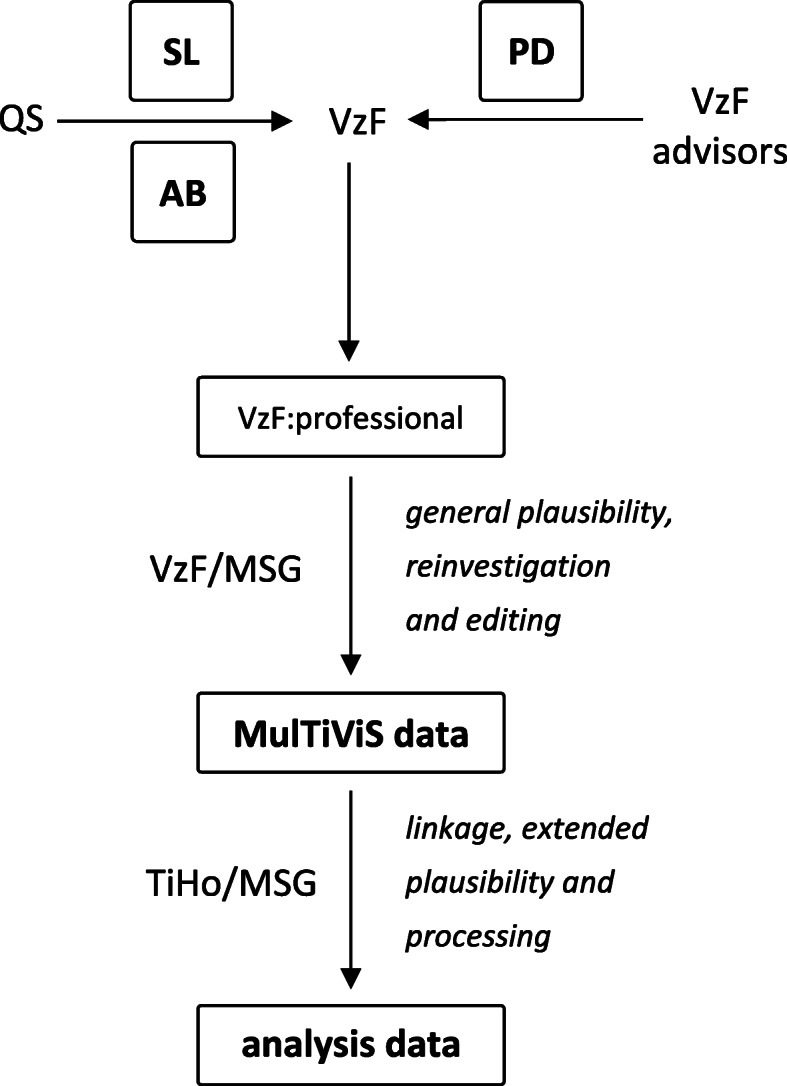
MulTiViS data management procedures (*MulTiViS* project on multivariate assessment of pig welfare, *QS* Quality Scheme for Food, *VzF* swine advisory service, *VzF:professional* processing software, *MSG* Marketing Service Gerhardy, *TiHo* University of Veterinary Medicine, Hannover, *SL* diagnostic data from slaughter, *AB* information on antibiotic usage, *PD* performance data)

All data sets were related to a specific time-span of each half-year from July 1, 2017 to June 30, 2019. Hence, we investigated four data sets for each data source. VzF collected the raw data and incorporated it into the company-owned data processing software “VzF:professional”. VzF and MSG conducted first analysis and editing steps as well as plausibility checks by reinvestigation of missing information and implausible values. Furthermore, new variables were established to build up the “MulTiViS dataset”. These data were transmitted to TiHo, which merged the datasets and checked for integration and statistical plausibility (Fig. 1).

### Definition of health scores

To assess the health status of pig herds, indicators from the animal-based measures were defined in the given data sets (Table 1), regarding German animal welfare discussions [6, 17, 18] and experiences of the project team. To use those indicators for health monitoring, they were transformed to specific scores that allow for distinguishing between different farms and time-periods. For this, the general approach proposed by Nienhaus et al. [16] was adapted. This method follows normalisation of skewed data via ordinary logarithm to a base of ten and a logit-transformation of prevalence data, respectively, to harmonise the information. Previously, 0-values were set to the half of the lowest value to avoid mathematical errors caused by division by zero. Afterwards, z-standardisation, i.e., z_i_ = [(x_i_ - $\overline{\mathrm{x}}$)/s] puts all variables on an equal scale with a mean of zero and a standard deviation of 1. Following this, negative values generally indicate an over-average good health status and positive values generally represent a health status inferior to the mean.

**Table 1:** Chosen indicators and health scores (*MOR* mortality score, *ADG* average daily weight gain score, *FCR* feed conversion ratio score, *TF* treatment frequency score, *RESP* respiratory lesions score, *EXT* exterior lesions score, *MANG* animal management score, *UDD* used daily doses, *FP* finishing pig place) with expert weights

Score	Indicator	Unit	Description	Expert weights^a^
**MOR**	mortality	%	dead and culled animals	-^b^
**ADG**	average daily gain	g	liveweight gain per day	-^b^
**FCR**	feed conversion ratio	kg/kg	feed per unit of liveweight gain	-^b^
**TF**	treatment frequency	UDD/FP	number of used daily doses per finishing pig places	-^b^
**RESP**	pneumonia	%	alteration of lung > 10%	5
	pleurisy	%	alteration of pleura > 10%	5
	pericarditis	%	alteration of pericardium	4
**EXT**	arthritis	%	inflammation of joint	3.5
	abscess	%	abscess	3
	ear lesions	%	necrosis/inflammation of ear	5
	tail lesions	%	necrosis/inflammation of tail	5
	dermal alterations	%	inflammation of skin	4
	bursitis	%	bursitis with > 5 cm in diameter	2.5
**MANG**	liver milk spots	%	alteration of liver by milk spots	4.5
	dermal damage	%	alteration through punch marks	5
	intestinal alteration	%	inflammation of intestines	1
	whole carcass condemnation	%	extensive alteration of carcass	1.5

^a^Adopted by Nienhaus et al. [16]

^b^No expert weights needed because of direct implementation to z-scores

From PD, we selected mortality, average daily gain and feed conversion ratio as health indicators and transformed them to the scores MOR, ADG and FCR, respectively. To ensure the same general interpretation, the ADG score was multiplied by − 1, because in contrast to the other indicators, high values are representing good performance.

To measure the use of antibiotics within a pig unit, a herd-specific treatment frequency was calculated by:

$$ Treatment\  fr\mathrm{e} quency=\frac{nUDD}{\# FP} $$

with nUDD = number of used daily doses = number of animals treated × number of days treated × number of active ingredients and # FP = number of finishing pig places. This definition is in line with the definition of the German Medicinal Products Act and recent scientific analyses [19, 20]. A transformation of the treatment frequency via natural logarithm and z-standardization led to TF score.

As SL-indicators, 13 assessments of carcass and organs from the QS meat inspection scheme (Table 1) were chosen. Although, since 2018, all slaughterhouses participating at the QS scheme work according to specific standards [13], it is known that there are still differences in the prevalence levels of recorded pathogenic lesions between the abattoirs [8, 21]. Hence, we defined a specific correction factor F_k_ for each abattoir k that compensates these differences. At the slaughterhouses, lesions were recorded for every single pig, which yields to 11917 different abattoir-herd-date combinations (batches). Batches of less than ten pigs were excluded. Furthermore, some farmers served more than one abattoir in the chosen six-month period. Hence, an adjusted herd-specific prevalence P_i_ for each pig unit i was defined as shown in the following formula:

$$ {P}_i=\sum \limits_k\left(\frac{n_{ik}}{\sum_{n_{ik}}}\times {P}_{ik}\times {F}_k\right) $$

with P_i_ = herd-specific prevalence of one indicator in half a year, n_ik_ = number of animals from pig unit i to abattoir k and P_ik_ = prevalence of pig unit i at abattoir k. Because the grades of pneumonia and pleurisy are documented in four categories, we decided to combine moderate (10–30%) and high (> 30%) alterations as positive records and merged slight (< 10%) and no alterations as negative records to report a unique prevalence. For the other SL-indicators, the original information of presence or absence were used. Following the approach of Nienhaus et al. [16], 13 SL-indicators were aggregated to scores, weighted by expert opinions (Table 1), to reduce complexity. A respiratory lesions score (RESP) was composed of pneumonia, pleurisy and pericarditis; an exterior lesions score (EXT) was composed of arthritis, abscess, ear lesions, tail lesions, dermal alterations and bursitis; and animal management score (MANG) was composed of liver milk spots, dermal damage, intestinal alteration and whole carcass condemnation.

### Aggregation to a total score

Finally, the health scores were aggregated to a total score (TOTAL) that should give a rough estimate of the “average” herd health status. Nienhaus et al. [16] used expert opinions to give the single health scores specific weights. However, we do not have information about *salmonella* status, but ADG and FCR instead. Coming from this, we both gave ADG and FCR the expert weight of *salmonella* status, which yielded the following formula:

$$ \mathrm{TOTAL}=\left(\left(5\times \mathrm{MOR}\right)+\left(2.5\times \mathrm{ADG}\right)+\left(2.5\times \mathrm{FCR}\right)+\left(3.5\times \mathrm{TF}\right)+\left(5\times \mathrm{RESP}\right)+\left(4\times \mathrm{EXT}\right)+\left(4.5\times \mathrm{MANG}\right)\right)/27 $$

To compare the method of Nienhaus et al. [16] with unweighted score aggregation, we opposed both score-rakings in a scatter plot.

### Temporal development of health scores in the study collective

As the defined health-scores are on a z-scale, this leads to a relative benchmarking. A z-value provides information about the health status of the respective farm compared to the others in the collective. To assess the variation of the health ranking of the pig herds during the study period, they were assigned to categories. These were based on classification of the collective in quarters, as follows:

- category 1: z-value ≤0.25-quartile,
- category 2: 0.25-quartile < z-value ≤0.5-quartile,
- category 3: 0.5-quartile < z-value ≤0.75-quartile,
- category 4: z-value > 0.75-quartile.

These categories were calculated for each score, each half-year and the IBW-class, respectively.

Thereupon it was assessed how the individual farms switched between the categories over the four half-years. Four categories were defined, depending on if the herds stayed in the upper, middle or lower 50% of the collective or if higher variations occurred.

All statistical evaluations mentioned were performed with SAS®, version 9.4.

## Results

### Stratification by IBW

**Fig. 2:**
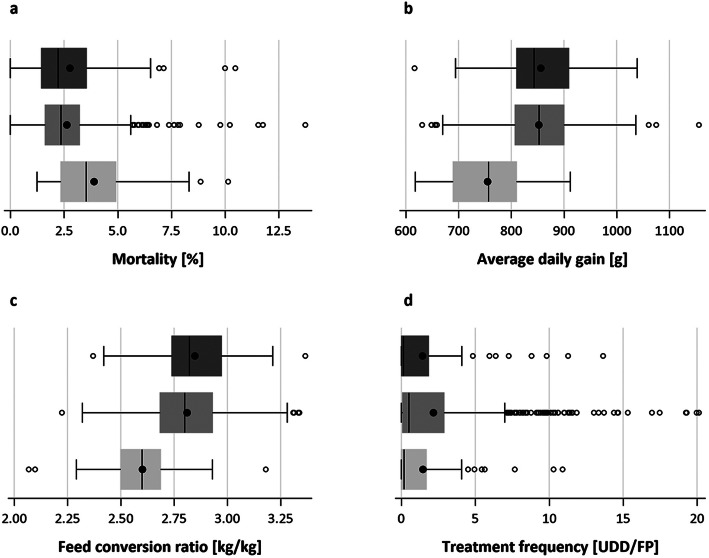
Variation of performance between different IBW-classes (1 = light (light grey), 2 = medium (grey), 3 = heavy (dark grey)) in mortality (**a**), average daily gain (**b**), feed conversion ratio (**c**) and treatment frequency (**d**) in the study collective with *n* = 736 (184 pig units × 4 half-years) in time-span from July 1, 2017 to June 30, 2019

Descriptive statistical evaluations showed that the mean of important indicators varies between classes of light or heavy IBW and the medium class, respectively (Fig. 2, Table 2). The results of the t-tests proved statistical significance of higher mortality (*p* <  0.0001) and lower average daily gain (p <  0.0001) and feed conversion ratio (p <  0.0001) for light class against medium class. For heavy class against medium class, treatment frequency was significantly lower (*p* = 0.0367).

**Table 2:** Means of four indicators mortality, average daily gain, feed conversion ratio and treatment frequency for different IBW-classes (1 = light, 2 = medium, 3 = heavy) and *p*-values of t-test

Indicator	Mean			P-Value t-test	
	Class 1	Class 2	Class 3	Class 1 vs. 2	Class 2 vs. 3
Mortality [%]	3.90	2.63	2.77	**< 0.0001**	0.5483
Average daily gain [g]	755.2	852.3	856.1	**< 0.0001**	0.6565
Feed conversion ratio [kg/kg]	2.60	2.81	2.85	**< 0.0001**	0.1067
Treatment Frequency [UDD/FP]	1.46	2.17	1.43	0.0744	**0.0367**

### Transformation of original health data

**Fig. 3:**
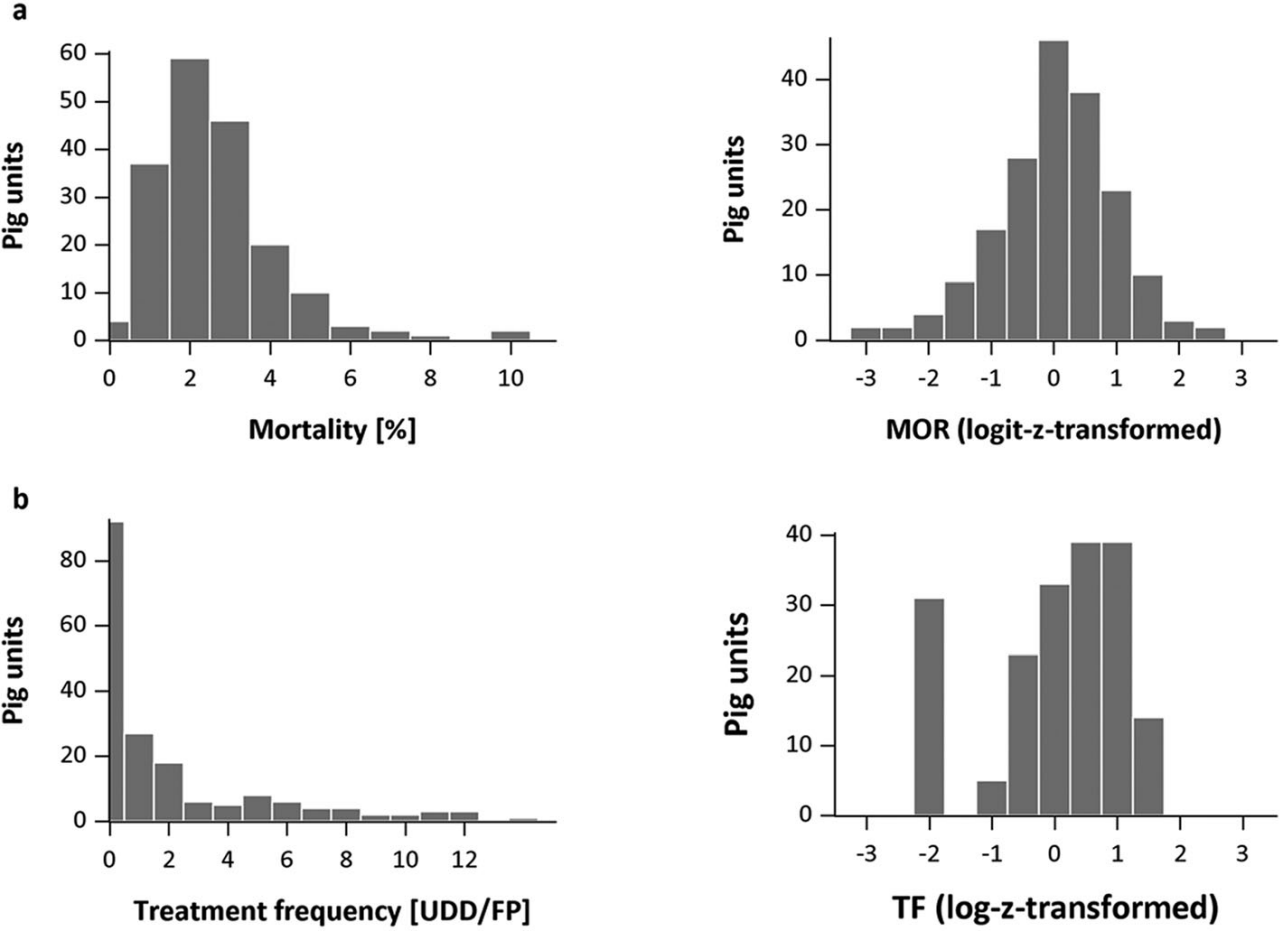
Distribution of untransformed and log(it)-z-transformed data for mortality (**a**) and treatment frequency (**b**) in the MulTiViS collective with *n* = 184 pig units in time-span from July 1, 2017 to December 31, 2017 (*MOR* mortality score, *TF* treatment frequency score, *UDD* used daily doses, *FP* finishing pig place)

In total, seven health scores (MOR, ADG, FCR, TF, RESP, EXT and MANG) and one total score were defined for 184 pig units and four half-years. The original data for ADG and FCR criteria appeared nearly normally distributed, and no substantial outlier was identified. In contrast, the untransformed data of MOR, TF and the SL-scores showed a skewed distribution. After transformation via natural logarithm to a base of ten for treatment frequency and logit-transformation for mortality and SL-indicators, they showed normal distribution and zero-inflation for variables with many zero values (Fig. 3).

**Fig. 4:**
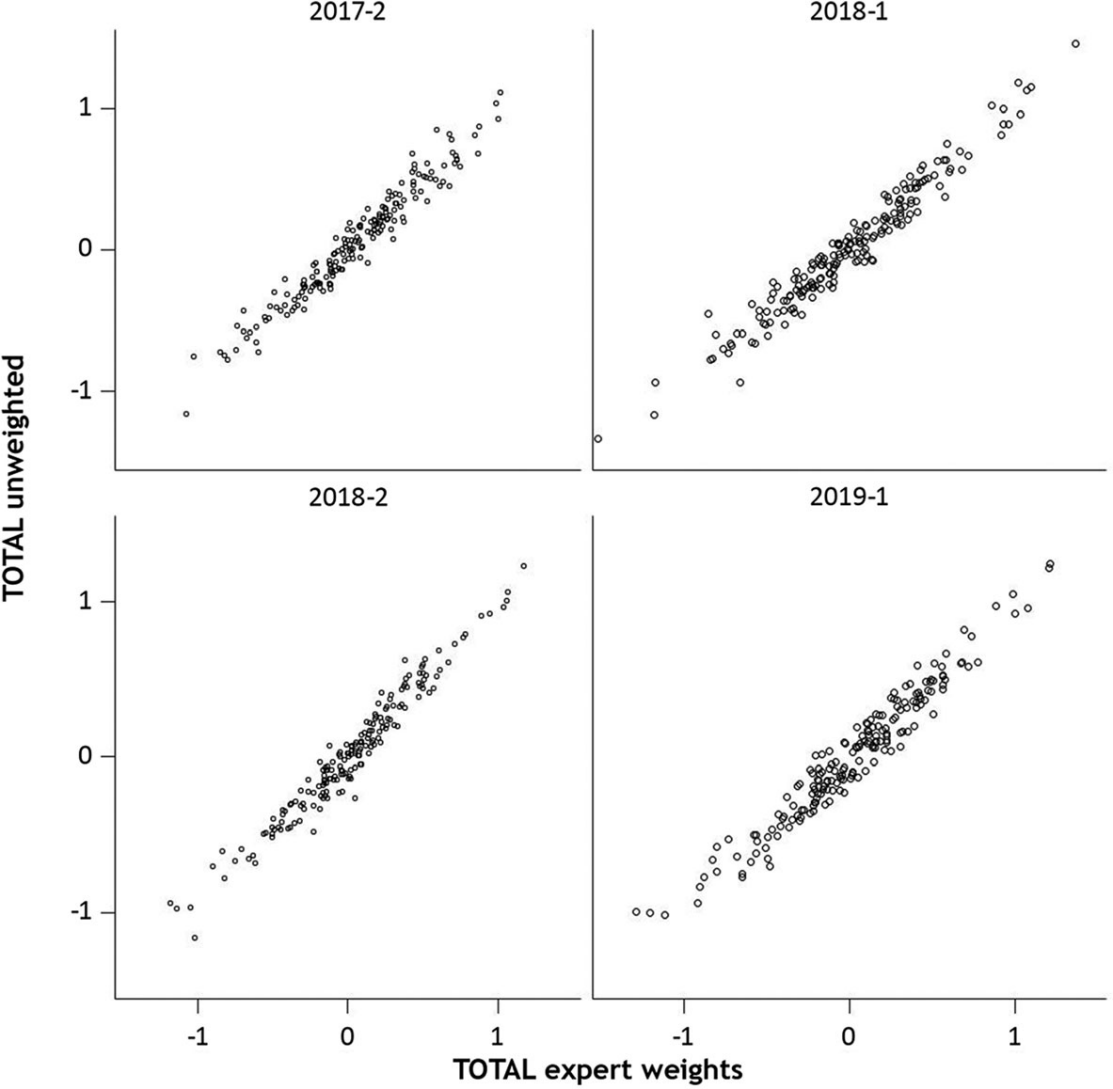
Comparison of ranking for total score (TOTAL) in four half-years, calculated with expert-weights and unweighted

The scatterplot comparing the two methods of calculating the total score shows in the four half-years that most pig units are at or near the diagonal and only a few outliers can be seen (Fig. 4). This means that ranking-position on z-scale does not differ substantially between the two scoring methods. Hence, we decided to adopt the expert weights from Nienhaus et al. [16].

### Distribution and temporal development of indicators

As most of the data were skewed, median instead of mean as descriptive parameter was used (Table 3). The following results refer to IBW-class 2, since the majority of pig units under study is in this class. Descriptive statistics for class 1 and 3 could be found in Additional file 1. The three PD indicators mortality, daily weight gain and feed conversion ratio varied very little over the time. The median mortality was between 2.26% and 2.40%, the median of average daily gain varied between 849 g and 860 g and feed conversion ratio was between 2.77 kg/kg and 2.81 kg/kg. A clear downward trend can be seen in the treatment frequency. It decreases from 0.72 UDD/FP in the first half-year to 0.46 UDD/FP in the last half-year. Descriptive measures of meat inspection data show that the variation over the four half-years was equally low for all SL-indicators. However, it is striking that classic indicators (pneumonia, pleurisy, pericarditis and liver milk spots) were found significantly more often than the new ones, which are only mandatory since 2018. Their median prevalence was below 1%. Pneumonia was found most frequently in the study period with a median prevalence between 8.52% and 10.74%, followed by pleurisy, pericarditis and liver milk spots. Ear lesions and dermal damage caused by handling occurred in less than half of the study collective in all four half-years.

**Table 3:** Median (P50) and Interquartile range (IQR) of indicators in four half-years from July 1, 2017 to June 30, 2019 for IBW-class 2 (n = 610, *UDD* used daily doses, *FP* finishing pig place)

Indicator	2017–2		2018–1		2018–2		2019–1	
	P50	IQR	P50	IQR	P50	IQR	P50	IQR
Mortality [%]	2.38	1.60	2.26	1.45	2.37	1.59	2.40	1.75
Average daily gain [g]	856	85	850	95	852	69	860	105
Feed conversion ratio [kg/kg]	2.81	0.20	2.80	0.26	2.81	0.24	2.77	0.30
Treatment frequency [UDD/FP]	0.72	2.90	0.49	2.92	0.55	3.12	0.46	2.61
Pneumonia [%]	10.74	8.91	10.34	9.26	8.52	8.90	9.64	11.33
Pleurisy [%]	4.54	7.44	4.15	8.58	3.74	5.52	4.50	8.99
Pericarditis [%]	3.00	2.26	2.97	2.76	3.15	2.69	3.92	2.93
Arthritis [%]	0.55	0.65	0.50	0.60	0.66	0.86	0.40	0.61
Abscess [%]	0.87	0.67	0.79	0.62	0.92	0.82	0.90	0.82
Ear lesions [%]	0.00	0.00	0.00	0.00	0.00	0.00	0.00	0.04
Tail lesions [%]	0.42	0.79	0.37	0.73	0.54	1.03	0.57	1.03
Dermal alterations [%]	0.07	0.20	0.12	0.28	0.15	0.22	0.08	0.20
Bursitis [%]	0.45	0.69	0.29	0.51	0.60	0.72	0.58	0.87
Liver milk spots [%]	3.89	6.08	3.40	4.62	3.51	5.79	3.16	5.28
Dermal damage [%]	0.00	0.00	0.00	0.00	0.00	0.00	0.00	0.00
Intestinal alteration [%]	0.47	0.58	0.45	0.64	0.38	0.59	0.27	0.62
Whole carcass condemnation [%]	0.09	0.20	0.13	0.25	0.11	0.23	0.08	0.16

### Development of health scores over time

One phenomenon in the temporal development of health benchmarking is that the majority of the collective has a variable health status over the four half-years under study, i.e. farms vary temporarily per score in ranking position. In contrast, very few herds remained in the middle 50% of the collective (category 2 or 3) over time.

To quantify this general effect, Table 4 shows the changes in benchmark categories for each health score and the total score. RESP seems to be the most constant parameter, because more than a quarter of the pig units were always found in the upper or lower half of the collective, respectively. In contrast, FCR is the score with the greatest variation. Approximately two-thirds of the pig units change their ranking position by more than one category during the study period.

**Table 4:** Categorisation of changes of the study collective (n = 184) for seven health scores (*MOR* mortality score, *ADG* average daily weight gain score, *FCR* feed conversion ratio score, *TF* treatment frequency score, *RESP* respiratory lesions score, *EXT* exterior lesions score, *MANG* animal management score) and one total score (TOTAL)

	Always category 1 or 2	Always category 2 or 3	Always category 3 or 4	Variable health status
	%	%	%	%
**MOR**	24.46	11.96	22.28	41.30
**ADG**	22.83	11.41	20.65	45.11
**FCR**	13.59	7.61	11.41	67.39
**TF**	20.65	14.13	19.02	46.20
**RESP**	28.80	11.41	26.63	33.15
**EXT**	14.67	10.33	14.67	60.33
**MANG**	23.37	13.59	22.83	40.22
**TOTAL**	21.20	12.50	21.20	45.11

## Discussion

The study aimed to connect different data sources from daily production processes along the supply chain for the purpose of a health monitoring programme of finishing pigs. Seven health scores and one total score were used to classify pig units by their health status: MOR, ADG, FCR, TF, RESP, EXT and MANG.

Combining seven individual scores into one total score has the advantage of providing a quick first impression of the health status. Furthermore, such a rough assessment is easier to handle for a monitoring system. However, such an overall score cannot indicate in which areas the individual pig units have potential for improvement. For this reason, it is imperative that the individual health scores are consulted as part of veterinary and agricultural advice.

### Sampled collective of pig units

Because the study is part of the process aiming to find methods for a national health monitoring programme, a high number of approximately 200 participating pig units was needed. However, because the collective of finishing pig herds in the study is based on both membership of VzF and willingness to participate, it may not be free of a selection bias.

Moreover, the location of the pig units has to be taken into account, as there are great regional differences concerning farm sizes and density of pig husbandry in Germany [22]. All participating herds were located in the northeast of Lower Saxony, which is a region with an extended density of pig husbandry compared with other German regions [23]. In November 2017, 4.3 million finishing pigs on 5100 farms were kept in that region [23]. Since the VzF population was not selected randomly and is located only in Lower Saxony, the results of the study are not purely representative of the population of finishing pig farms in Germany. However, due to VzF as a consultant for typical pig farming in Germany, the sample is seen as a foundation for a study design for a national monitoring programme because it provides insight into the general suitability of secondary data usage for welfare scoring.

### Stratification by IBW-classes

The IBW of pigs when installed has impact on several health parameters. Results of t-test showed that for light class-1-pig herds mortality is higher and average daily gain is lower than in class-2 herds. One possible explanation is that younger piglets are more susceptible to health problems because their immune system is not yet fully developed and that they are reared for a longer period, which leads to higher mortality. Furthermore, younger piglets have relatively better feed conversion ratio than older ones. However, the growth curve of weight gain per kilogram live weight has its peak in the later phase of the finishing period [24]. This is a possible explanation for lower average daily weight gain of pig units in class 1, as the calculation of daily gain takes into account the complete finishing period and therefore, in the case of class-1 herds, also the lower values from the initial period.

The restricted usage of antibiotics in class-3 herds could be caused by the fact, that the piglets are stalled in with higher age because they had a pre finishing phase and therefore a shorter time that is depicted in the study data. These animals have usually overcame the typical “childhood diseases” and therefore need less antibiotic treatment.

### Indicators for health scoring

This study concentrated on health- and performance-related items as they can be easily quantified and are often harmonised and captured within daily processes along the supply chain. The selection of specific health indicators was based on expertise of the project teams and results of various national and international studies that tried to quantify pig health and welfare.

In Germany, the “Kuratorium für Technik und Bauwesen in der Landwirtschaft e.V.” (KTBL) conducted an expert survey to define welfare indicators with high validity and reliability under the usual conditions of the German production system. To keep the economic effort manageable, they preferred indicators built from existing databases [25]. For the same reason, we took the KTBL indicators mortality, average daily weight gain, feed conversion ratio, treatment frequency and assessments of meat inspection into account [6]. KTBL outlines that these data could be a hint for possible welfare problems [25].

In 2009, Dickhaus et al. [18] tried to quantify the health status of pig herds with a herd health score (HHS), which includes information about the mortality rate, the frequency of pathological assessments in carcasses and organs, an animal-treatment-index and the duration of the finishing period. It was noted that the combination of this information is suitable for benchmarking systems as a tool for improving animal health and welfare. With the exception of the fourth point (duration of finishing period), these variables are the same as those selected for our study. Due to the lack of information concerning the duration of the finishing period, we decided to use average daily gain and feed conversion ratio instead.

Pandolfi et al. [26] merged different data sources to assess the interconnections between biosecurity, health, welfare and performance in commercial pig farms in Great Britain. They had performance data (average daily gain, mortality and feed conversion ratio) from farm visits, welfare information from the Real Welfare programme and lesions recorded at the abattoir from the British Pig Health Scheme. Additionally, they collected information about external and internal biosecurity during farm visits to examine the association between biosecurity and welfare outcomes. The Real Welfare Scheme is a programme of the Agricultural and Horticultural Development Board (AHDB) within the frame of quality assurance (Red Tractor) and covers 95% of the production [27]. It contains welfare indicators that are derived from on-farm data related to the prevalence of pigs that would benefit from removal to hospital pens, lame pigs, pigs with tail damage, pigs with body marks and environmental enrichment provision and use [27].

Overall, it may be stated that the variables chosen for scoring are in line with the scientific literature and could be collected in daily processes. However, as the data were not originally gathered for the purpose of health and welfare monitoring, secondary data use has to be addressed in more detail under the conditions of the German production system.

### Secondary data analysis

As this investigation is an observational study, it was targeted to examine the usability of already existing information sources for monitoring, and only secondary data drawn from processes in the supply chain was used. The data were collected within the scope of consulting (PD) and quality assurance (AB, SL) but not with the aim of an interdependent analysis or to use it for combined health monitoring.

A striking advantage of secondary data usage is the lack of effort needed to acquire the data. Therefore, it requires fewer economic and personnel resources without any additional burden for the farmers.

However, the use of secondary data is not straightforward as they are collected for another purpose. The data need to be cleaned and checked for both integration and plausibility, which requires extra work. Due to their internal and external plausibility as well as association structures, not all variables can be used. Implausible and missing values might occur. On the one hand, these processes are linked to the data source, and on the other hand, they are connected to the purpose of the analyses, i.e., in our case, the scoring of animal health [28]. Therefore, a source-by-source discussion is necessary.

#### Biological and economic performance data

In contrast to AB and SL, for which strict documentation is mandatory by law, general performance data (PD) on herd level are not standardised on a national level in Germany. Rules for a harmonisation of these data are rare. Only the documentation of mortality is required by law. However, such data collection is requested or already implemented by various instances, for example, “vit” [29], that offers a harmonised data approach for its members or, much stricter, the chamber of veterinarians, that demands a standardised, routine documentation of performance data [30]. However, pig units connected to consulting services such as VzF work with their own standards based on the suggestions mentioned above.

For MulTiViS, PD was constructed from the internal VzF database, which is merged from data, the pig units documented due to different regulations as well as from different optional data during the production process. For this, agricultural advisors collected basic claims data of the pig units as well as variable data of the production process. The latter was aggregated by time as well as between the pig units’ compartments. For categorical data, if expressions vary in different compartments within the pig unit, VzF has defined the subclasses according to the majority principle. For continuous data, usual averages, weighted by the number of animals, were used. Therefore, on the one hand, these data are prone to an information bias, especially if the pig units hold a huge variety of different compartments. On the other hand, continuous data about mortality, average daily gain and feed conversion ratio are usually under strict farmer control, which generally avoids serious bias.

#### Information on antibiotic usage

Since their collection is related to the mandatory documentation in the German Medicinal Products Act, register data on antibiotic usage are available for all finishing pig units of a certain size. In addition, AB data from pig units participating in the QS quality programme, are available for pig units of all sizes. Because the QS system covers 95% of the entire German pork production system, the information on AB is close to covering the general target population [10].

Despite this advantage, the QS system has pitfalls. First, information about the indication of antibiotic treatment is usually lacking. Therefore, a direct link to animal health aspects is not possible. Furthermore, bias may occur from the veterinary practices because the methods and intensity of antibiotic treatments differ. Another problem is information bias of AB as these data are generally used for farm consulting. Therefore, plausibility checks were only performed if the treatment frequency is above specific cut-off values in the benchmarking system. Furthermore, plausibility checks were only executed for general amounts but not for substances in detail.

The general usability of AB as health indicator should also be critically reviewed, as the association of extended antimicrobial treatment with diseased animals (poor health status) is often broken twice. On one hand, (high) antibiotic usage could also be associated with good animal health because it is used for the purpose of curing. On the other hand, farmers may avoid antibiotic therapy to keep from being benchmarked but that could cause poor animal health.

#### Meat inspection data

The general usability of slaughterhouse data for animal health and welfare purposes in Germany is well discussed [21, 31]. Although the gathering of these data is formally harmonised, in daily practice, there is a lack of standardisation between abattoirs and inspectors. This leads to differences of the prevalence levels on the temporal scale as well as between slaughterhouses. Therefore, only some studies found a relationship between respiratory lesions at the abattoir and the respiratory health and performance of living pigs [32, 33]. To compensate for this phenomenon, we have made a specific correction to the individual prevalence data.

As the collection of findings according to the present QS scheme has only been established mandatory since 2018, the reporting of the newly added lesions is therefore correspondingly non-standardised. This is manifested by a prevalence of below 1% and a skewed distribution which hence limits the statistical usability.

However, information of meat inspection from QS is the first nationwide data collection in Germany. It covers approximately 95% of the entire German pork production system, which corresponds to information of approximately 30000 pig units and 200 abattoirs in one system. For that reason, the use of these data has to be stated as crucial.

### Relative benchmarking of health scores

Because the original data are on different scales, the indicators were standardised via z-transformation to health scores, following the approach from Nienhaus et al. [16]. That implies each pig unit is assigned a certain rank, reflecting its relative position within the distribution of the study collective. If the collective of pig units was changed, i.e. between different time-spans, a shift of the individual z-value would be possible. Hence, health-scores based on z-values only allow conclusions to be drawn about the health status of a pig herd in comparison to the other herds under study. However, there is always an incentive for improvement if a pig unit has a high z-value (which stands for relative poor health status) or if the z-value is worse than in the previous half-year. Either the own health status declined or the average performance in the collective improved. Another advantage of such relative scoring method is that no absolute threshold needs to be defined and that trends over time can be offset.

This strategy is in line with other benchmarking systems. As an example, the monitoring programme of antibiotics due to the 16th amendment of the German Medicinal Products Act is using the 50%- and the 75%-percentile of the entire distribution of pig units as benchmarks. Twice a year, these measures are calculated, so the critical thresholds change every 6 months.

### Temporal development of health status

All the indicators examined except the TF showed only moderate fluctuation over time. For the TF, however, a clear downward trend could be identified. The increasing problem of antimicrobial resistances has led to a strong focus on the use of antibiotics in livestock production. Both nationally and internationally, there are calls for a reduction in the use of antibiotics in farm animals [34–36]. This is reflected in the decreasing TF in the study farms.

Another temporal factor is seasonality, which has an influence on many aspects of health, e.g. mortality or feed conversion ratio [37, 38]. However, as the available data in the study was collected on a half-year basis and include parts of the warm and cold seasons each, rough trends can be balanced out. This can be seen in Table 3, where is shown that there are no strong fluctuations between the half-years.

For relative benchmarking with health scores, it can be stated that changing of benchmark categories may be attributed to two different causes: firstly, the health status of a certain farm improved or declined; secondly, the health status remained the same but the other pig herds in the collective performed better or worse.

The results from Table 4 showed, that most pig units have varied strongly in their benchmark position. This again underlines the advantage of a relative benchmarking system, which takes account of such fluctuations.

It can also be noted that the pig herds that did not fluctuate that much were found either in the upper or the lower half of the collective. Constantly moderate farms, that were steadily in category 2 or 3 over time, occurred very seldom. This suggests that the health status of averagely performing farms usually changes in one direction or another. Reasons for this could be, i.e., veterinary advice or special events such as a vaccination breakthrough or disease outbreak.

Monitoring the scores over time can provide an indication of whether an individual farm is experiencing sustained animal health problems. If, for example, it remains in the worst category over all four half-years, it either has long-term difficulties with health management or it cannot keep up with the upward trend of the other farms. Both, however, highlight the need for sustainable veterinary or agricultural advice. If, on the other hand, a pig unit often changes category, this is probably more likely to be due to constant fluctuations in the collective or short-term changes or spontaneous events in health management.

## Conclusion

The study proves that routine data from existing databases of the German pork production chain are usable for health monitoring. However, it has to be stated, that preparatory editing steps are crucial. Furthermore, we suggest stratification by IBW of piglet, because this has impact on important health indicators. A total score could give a rough estimate of a herd health status but should be complemented by specific health scores in the frame of veterinary and agricultural advising. It was also shown that relative benchmarking can be used to depict fluctuations in the composition of the target collective and temporal conditions, without having to define absolute thresholds. Additionally, it is also a constant incentive for individual farms to improve their health status. Evaluating the health scores over time can also show which farms have sustainable improvement potential.

## Supplementary Information

**Additional file 1.** Descriptive statistics of indicators for IBW-classes 1, 2 and 3.

## Data Availability

The data were collected on an individual basis from farmers and slaughterhouses. Each participant gave written consent with the understanding that data would not be transferred to a third party. Therefore, any data transfer to interested persons is not allowed without an additional formal contract. Data are available to qualified researchers who sign a contract with the University of Veterinary Medicine Hannover and VzF. This contract will include guarantees to the obligation to maintain data confidentiality in accordance with the provisions of the German data protection law. Currently, there exists no data access committee or another body that could be contacted for the data. However, for this purpose, a committee will be founded. This future committee will consist of the authors as well as members nominated by the University of Veterinary Medicine Hannover and the VzF. Interested cooperative partners, who are able to sign a contract as described above, may contact: Prof. Dr. Lothar Kreienbrock, Department of Biometry, Epidemiology and Information Processing,University of Veterinary Medicine, Hannover, Buenteweg 2, 30559 Hannover, Email: lothar.kreienbrock@tiho-hannover.de

## Abbreviations

ADG: Average daily weight gain score
EXT: Exterior lesions score
FCR: Feed conversion ratio score
FP: Finishing pig place
IBW: Initial body weight
IQR: Interquartile range
MANG: Animal management score
MOR: Mortality score
nUDD: Number of used daily doses
PD: Performance data
QS: Quality Scheme for Food
RESP: Respiratory lesions score
SL: Diagnostic data from slaughter
TF: Treatment frequency score
