# Supplementary material for: Health monitoring of finishing pigs by secondary data use – a longitudinal analysis
Source: Porcine Health Manag. 2021 Feb 24;7:20. doi: 10.1186/s40813-021-00197-z (PMC7903635; doi:10.1186/s40813-021-00197-z)
Supplement: Supplementary file 1 — Additional file 1. Descriptive statistics of indicators for IBW-classes 1, 2 and 3. [file 40813_2021_197_MOESM1_ESM.pdf]

Descriptive statistics of indicators for IBW classes 1, 2 and 3

Table A Median (P50) and Interquartile range (IQR) of indicators in four half-years from July 1, 2017 to June 30, 2019 for Class 1 (n = 51)

Indicator	2017-2		2018-1		2018-2		2019-1	
	P50	IQR	P50	IQR	P50	IQR	P50	IQR
Mortality [%]	3.08	1.36	3.65	3.09	3.26	2.01	4.51	2.28
Average daily gain [g]	725	125	717	100	791	114	749	90
Feed conversion ratio [kg/kg]	2.63	0.23	2.65	0.26	2.56	0.16	2.58	0.20
Treatment frequency [UDD/FP]	0.35	2.88	0.08	1.89	0.18	1.27	0.24	0.85
Pneumonia [%]	10.33	6.86	10.46	5.38	7.46	5.77	7.17	11.74
Pleurisy [%]	3.39	6.10	2.43	9.54	1.70	5.39	2.64	10.98
Pericarditis [%]	2.77	2.08	2.35	0.86	2.43	2.39	3.36	1.70
Arthritis [%]	0.26	1.13	0.35	0.76	0.62	1.06	0.23	0.48
Abscess [%]	0.78	1.30	0.80	0.64	0.75	0.94	0.93	0.95
Ear lesions [%]	0.00	0.00	0.00	0.00	0.00	0.00	0.00	0.00
Tail lesions [%]	0.16	0.79	0.58	1.19	1.02	1.20	1.13	1.41
Dermal alterations [%]	0.05	0.20	0.00	0.10	0.11	0.39	0.05	0.34
Bursitis [%]	0.29	0.91	0.22	0.47	0.61	0.59	0.43	0.56
Liver milk spots [%]	2.80	4.80	2.91	4.17	3.09	3.59	4.28	3.93
Dermal damage [%]	0.00	0.02	0.00	0.02	0.00	0.00	0.00	0.00
Intestinal alteration [%]	0.42	0.51	0.45	0.45	0.50	0.18	0.45	0.42
Whole carcass condemnation [%]	0.00	0.10	0.02	0.13	0.06	0.36	0.09	0.25

Table B Median (P50) and Interquartile range (IQR) of indicators in four half-years from July 1, 2017 to June 30, 2019 for Class 2 (n = 610)

Indicator	2017-2		2018-1		2018-2		2019-1	
	P50	IQR	P50	IQR	P50	IQR	P50	IQR
Mortality [%]	2.38	1.60	2.26	1.45	2.37	1.59	2.40	1.75
Average daily gain [g]	856	85	850	95	852	69	860	105
Feed conversion ratio [kg/kg]	2.81	0.20	2.80	0.26	2.81	0.24	2.77	0.30
Treatment frequency [UDD/FP]	0.72	2.90	0.49	2.92	0.55	3.12	0.46	2.61
Pneumonia [%]	10.74	8.91	10.34	9.26	8.52	8.90	9.64	11.33
Pleurisy [%]	4.54	7.44	4.15	8.58	3.74	5.52	4.50	8.99
Pericarditis [%]	3.00	2.26	2.97	2.76	3.15	2.69	3.92	2.93
Arthritis [%]	0.55	0.65	0.50	0.60	0.66	0.86	0.40	0.61
Abscess [%]	0.87	0.67	0.79	0.62	0.92	0.82	0.90	0.82
Ear lesions [%]	0.00	0.00	0.00	0.00	0.00	0.00	0.00	0.04
Tail lesions [%]	0.42	0.79	0.37	0.73	0.54	1.03	0.57	1.03
Dermal alterations [%]	0.07	0.20	0.12	0.28	0.15	0.22	0.08	0.20
Bursitis [%]	0.45	0.69	0.29	0.51	0.60	0.72	0.58	0.87
Liver milk spots [%]	3.89	6.08	3.40	4.62	3.51	5.79	3.16	5.28
Dermal damage [%]	0.00	0.00	0.00	0.00	0.00	0.00	0.00	0.00
Intestinal alteration [%]	0.47	0.58	0.45	0.64	0.38	0.59	0.27	0.62
Whole carcass condemnation [%]	0.09	0.20	0.13	0.25	0.11	0.23	0.08	0.16

Table C Median (P50) and Interquartile range (IQR) of indicators in four half-years from July 1, 2017 to June 30, 2019 for Class 3 (n = 75)

**Indicator**	**2017-2**		**2018-1**		**2018-2**		**2019-1**	
	**P50**	**IQR**	**P50**	**IQR**	**P50**	**IQR**	**P50**	**IQR**
Mortality [%]	1.91	1.58	2.08	2.69	2.68	1.73	2.24	2.23
Average daily gain [g]	847	100	838	124	846	76	841	127
Feed conversion ratio [kg/kg]	2.89	0.22	2.86	0.27	2.82	0.11	2.72	0.26
Treatment frequency [UDD/FP]	0.13	1.14	0.08	0.35	0.29	1.81	0.29	2.35
Pneumonia [%]	11.66	9.72	12.33	6.33	12.23	6.94	11.17	19.60
Pleurisy [%]	4.39	7.26	5.24	15.99	7.74	7.41	5.98	17.57
Pericarditis [%]	4.64	4.37	3.84	3.02	4.59	3.38	5.24	5.75
Arthritis [%]	0.77	0.92	0.51	0.90	0.42	0.64	0.40	0.43
Abscess [%]	1.17	0.52	1.29	1.20	1.21	0.58	1.19	0.92
Ear lesions [%]	0.00	0.00	0.00	0.00	0.00	0.00	0.00	0.00
Tail lesions [%]	0.28	0.69	0.53	1.98	0.58	0.69	0.14	0.66
Dermal alterations [%]	0.15	0.23	0.07	0.26	0.08	0.21	0.05	0.44
Bursitis [%]	0.23	0.43	0.31	0.83	0.65	0.82	0.59	0.66
Liver milk spots [%]	6.55	8.52	4.63	4.09	6.33	5.90	4.26	2.87
Dermal damage [%]	0.00	0.00	0.00	0.04	0.00	0.00	0.00	0.00
Intestinal alteration [%]	0.61	1.21	0.44	0.56	0.69	0.59	0.89	0.68
Whole carcass condemnation [%]	0.05	0.18	0.18	0.36	0.16	0.22	0.16	0.24
